# Expression of *Flavone Synthase II* and *Flavonoid 3′-Hydroxylase* Is Associated with Color Variation in Tan-Colored Injured Leaves of Sorghum

**DOI:** 10.3389/fpls.2016.01718

**Published:** 2016-11-21

**Authors:** Hiroshi Mizuno, Takayuki Yazawa, Shigemitsu Kasuga, Yuji Sawada, Hiroyuki Kanamori, Yuko Ogo, Masami Yokota Hirai, Takashi Matsumoto, Hiroyuki Kawahigashi

**Affiliations:** ^1^Agrogenomics Research Center, National Institute of Agrobiological SciencesTsukuba, Japan; ^2^Institute of Crop Science, National Agriculture and Food Research OrganizationTsukuba, Japan; ^3^Faculty of Agriculture, Shinshu UniversityNagano, Japan; ^4^RIKEN Center for Sustainable Resource ScienceYokohama, Japan

**Keywords:** apigenin, apigeninidin, FNR, FNSII, F3′H, luteolin, luteolinidin, RNA-seq

## Abstract

Sorghum (*Sorghum bicolor* L. Moench) exhibits various color changes in injured leaves in response to cutting stress. Here, we aimed to identify key genes for the light brown and dark brown color variations in tan-colored injured leaves of sorghum. For this purpose, sorghum M36001 (light brown injured leaves), Nakei-MS3B (purple), and a progeny, #7 (dark brown), from Nakei-MS3B × M36001, were used. Accumulated pigments were detected by using high-performance liquid chromatography: M36001 accumulated only apigenin in its light brown leaves; #7 accumulated both luteolin and a small amount of apigenin in its dark brown leaves, and Nakei-MS3B accumulated 3-deoxyanthocyanidins (apigeninidin and luteolinidin) in its purple leaves. Apigenin or luteolin glucoside derivatives were also accumulated, in different proportions. Differentially expressed genes before and after cutting stress were identified by using RNA sequencing (RNA-seq). Integration of our metabolic and RNA-seq analyses suggested that expression of only *flavone synthase II (FNSII)* led to the synthesis of apigenin in M36001, expression of both *FNSII* and *flavonoid 3′-hydroxylase (F3′H)* led to the synthesis of apigenin and luteolin in #7, and expression of both *flavanone 4-reductase* and *F3′H* led to the synthesis of 3-deoxyanthocyanidins in Nakei-MS3B. These results suggest that expression of *FNSII* is related to the synthesis of flavones (apigenin and luteolin) and the expression level of *F3′H* is related to the balance of apigenin and luteolin. Expression of *FNSII* and *F3′H* is thus associated with dark or light brown coloration in tan-colored injured leaves of sorghum.

## Introduction

Sorghum (*Sorghum bicolor* L. Moench) exhibits various leaf color changes in response to disease, injury by insects, or wounding (Doggett, 1988). Leaves can turn purple, red, orange, or tan (brown), or in-between colors. The color depends on the sorghum cultivar. For example, the leaves of Nakei-MS3B and JN43 turn purple; those of bmr-6 turn reddish orange; those of BTx623 turn orange (Mizuno et al., 2014); and those of M36001, JP501, JP43800, JP588, JP43764, and Greenleaf turn tan (brown; Kawahigashi et al., 2016). Moreover, it is possible to distinguish light or dark brown colorations in plants with tan colors (Doggett, 1988). The tan-colored sorghums accumulate relatively high levels of flavones (apigenin and luteolin) than red/purple colored sorghums (Siame et al., 1993; Dykes et al., 2009, 2011). Colored pigments are accumulated in response to infection with the fungi *Bipolaris sorghicola* (Kawahigashi et al., 2011; Mizuno et al., 2012), *Colletotrichum sublineolum* (Snyder and Nicholson, 1990), *Cochliobolus heterostrophus* (Aguero et al., 2002), *Sporisorium reilianum* (Zuther et al., 2012) or to wounding stress (Mizuno et al., 2014). Pigment accumulation is considered to enhance resistance to pathogen infection (Hipskind et al., 1990; Snyder and Nicholson, 1990; Lo et al., 1999; Clifford, 2000; Kawahigashi et al., 2011; Zuther et al., 2012).

The *P* locus is one of the loci responsible for sorghum coloration (purple, red, or orange) and is dominant to *p* (tan; Doggett, 1988). The protein encoded by the *P* gene is flavanone 4-reductase (FNR), which is responsible for the synthesis of 3-deoxyanthocyanidins (Kawahigashi et al., 2016). The variation of purple-red-orange is explained by the balance of two 3-deoxyanthocyanidins (luteolinidin and apigeninidin; Mizuno et al., 2014). The balance is controlled by the expression level of *flavonoid 3′-hydroxylase (F3′H)*, which encodes a protein that hydroxylates the 3′ position of the B-ring of naringenin to produce a precursor of luteolinidin (Shih et al., 2006).

Sorghums with a tan injury response have non-functional *FNR* alleles (*p*). The *FNR* gene of JP501 and JP43800 has an insertion in the coding region; those of Greenleaf, JP588, and JP43764 have a Cys252Tyr amino acid substitution and encode unstable protein (Kawahigashi et al., 2016). Sorghums with a tan injury response have lower levels of flavan-4-ols (precursors of apigeninidin and luteolinidin—apiforol and luteoforol, respectively) than do sorghums with a purple or red injury response (Dykes et al., 2005); they are thus unable to synthesize 3-deoxyanthocyanidins. What genes are responsible for the coloration and color variation in sorghums with a tan (light or dark brown) injury response? These sorghums may show activation of an alternative pathway independent of 3-deoxyanthocyanidin synthesis.

Here, we aimed to identify key genes for the light brown and dark brown color variations in sorghum leaves with a tan injury response. For this purpose, we used sorghum Nakei-MS3B (purple response), M36001 (light brown response), and progeny #7 (dark brown response) derived from Nakei-MS3B × M36001. We performed a metabolic analysis to identify the accumulated pigments and an RNA sequencing (RNA-seq) analysis to identify the genes expressed in each line. Our data suggested that flavone synthase II (FNSII) pathways were activated to synthesize flavones (apigenin and luteolin) in sorghum with a tan response, and that levels of F3′H production changed the balance of the two flavones, thus resulting in their color response variation. We also discuss the differences in gene structure and gene expression networks determining each secondary metabolic pathway for coloration of sorghum leaves.

## Materials and Methods

### Plant Materials and Determination of Accumulated Pigments

Sorghum Nakei-MS3B (purple response), M36001 (light brown response), and progeny #7 (dark brown response) derived from Nakei-MS3B × M36001 were used. For the plant color test, F_2_ populations were grown at Shinshu University in Nagano, Japan, in 2011. At Tsukuba, Ibaraki, Japan, in 2012, the F_3_ populations were grown and subjected to mRNA-seq analysis. Details of the materials used have been given previously (Mizuno et al., 2014). Accumulated pigments were quantified by using HPLC at an absorbance of 475 or 290 nm (Ogo et al., 2013). Metabolic analysis of the accumulated pigments in each line was performed by using LC-MS/MS and standard authentic compounds (Sawada et al., 2009).

### RNA-seq

Four days after cutting stress, the edges of sorghum leaf strips that exhibited color changes in response to injury were collected. As controls, leaf strips immediately after cutting stress were also collected. To extract RNA, five biological replicates were collected, immediately frozen in liquid nitrogen, and mixed to minimize the effect of transcriptome unevenness among plants. RNA quality was calculated with a Bioanalyzer 2100 algorithm (Agilent Technologies, Palo Alto, CA, USA); high-quality (RNA integrity number >8) RNA was used. The protocol used for extraction of RNA and sequencing with an Illumina GAIIx sequencer (Illumina, San Diego, CA, USA) has been described previously (Mizuno et al., 2010). Reads were deposited in the DDBJ (DNA Data Bank of Japan) Sequence Read Archive (Accession No. DRA001265).

### Quantitative RT-PCR (qRT-PCR)

The same RNA material was shared for use in the Illumina RNA-seq and Quantitative RT-PCR (qRT-PCR) analysis. First-strand cDNA was synthesized from RNA (1 μg) in a 20-μl reaction mixture with a TaKaRa RNA PCR kit (AMV) v. 3.0 (TaKaRa Bio, Inc.). PCR was performed with initial denaturation at 98° for 2 min; 42 cycles of 98° for 10 s, 60° for 10 s, and 68° 30 s. qRT-PCR was carried out using Mx3000P (Stratagene Products Division, Agilent Technologies) with KOD SYBR^®^ qPCR Mix (Toyobo) according to the manufacturer’s recommendations and analyzed based on the delta-delta-Ct method. The gene transcripts were amplified with each specific primer pair (Supplementary Table S2). The value for each genes was normalized using SbActin (Sb03g040880) as an internal standard.

### Bioinformatics

Trimming of low-quality nucleotides (<Q15) from both the 5′- and the 3′-ends and of adaptors was performed by using Cutadapt version 1.0^1^. Bowtie 2 version 2.0.0 beta6 (Langmead and Salzberg, 2012) was used to align the reads against sorghum rRNA sequences (Ouyang and Buell, 2004); aligned reads were removed. Reads were aligned to the sorghum reference genome of BTx623 (Paterson et al., 2009) by using Bowtie 2, SAMtools version 0.1.18 (Li et al., 2009), and TopHat version 2.0.4 (Kim et al., 2013). RPKM (Reads Per Kilobase of exon model per Million mapped reads) values were calculated for each transcript annotated in Phytozome version 9 (Goodstein et al., 2012) or in non-annotated gene models constructed by using Cuﬄinks version 2.0.0 (Trapnell et al., 2010). Heatmaps were generated by using R^2^ package gplots version 2.10.1, with the RPKM of each gene model and the relative (after/before cutting stress) ratio of RPKM or that of the accumulated pigments determined by LC-MS/MS.

## Results

### Detection of Accumulated Pigments

**FIGURE 1:**
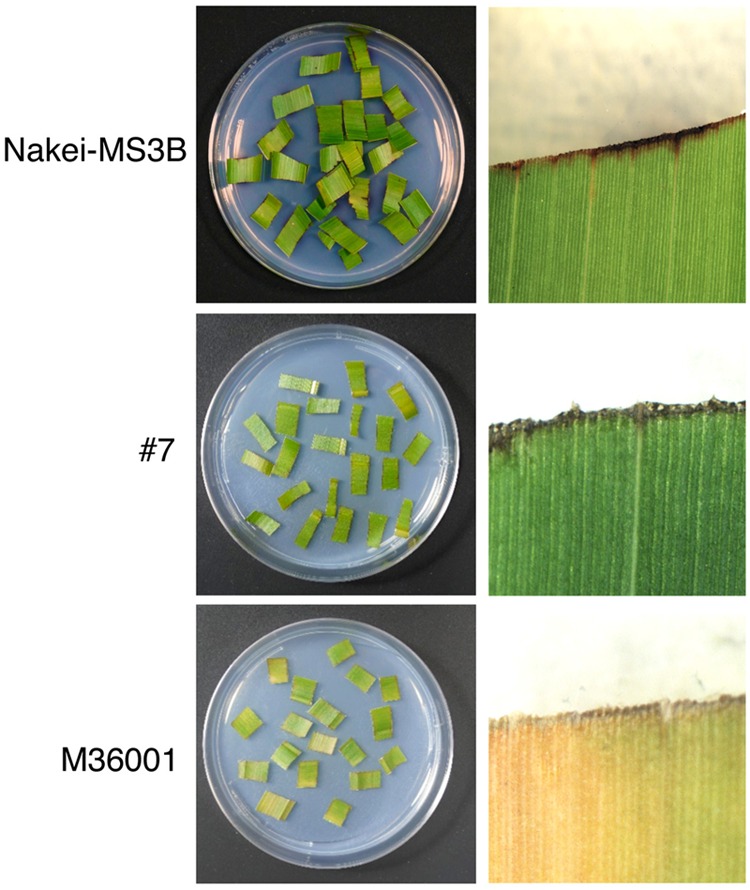
**Coloration of sorghum leaves after cutting stress.** Cut leaves on agarose gel (left) and enlarged images (right) are shown. Nakei-MS3B **(Top)** turns purple at the cut edge of each leaf fragment, but M36001 **(Bottom)** turns light brown. The F_1_ progeny #7 **(Middle)** of Nakei-MS3B × M36001 turns dark brown.

**FIGURE 2:**
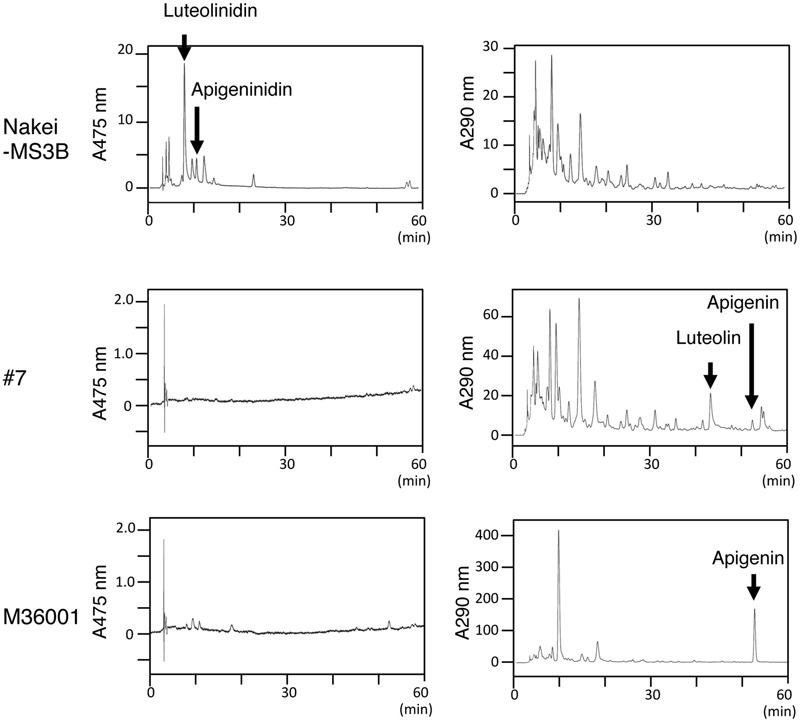
**Pigments accumulated after cutting stress.** Leaves were left for 4 days after cutting stress. Accumulated pigments were analyzed by HPLC at absorbances (A) of 475 or 290 nm. Nakei-MS3B accumulated 3-deoxyanthocyanidins (apigeninidin and luteolinidin), but #7 and M36001 did not. M36001 accumulated only apigenin, whereas line #7 accumulated both apigenin and luteolin.

Sorghum leaves exhibited various colors in response to cutting stress. Nakei-MS3B leaves had a purple edge, M36001 had a light brown edge, and progeny #7 from a cross between Nakei-MS3B and M36001 had a dark brown edge (**Figure 1**). These accumulated pigments were analyzed by using high-performance liquid chromatography (HPLC). Nakei-MS3B accumulated the 3-deoxyanthocyanidins luteolinidin and (a small amount of) apigeninidin in its purple region after cutting stress, whereas #7 and M36001 did not (**Figure 2**, absorbance [A] 475 nm). #7 accumulated both luteolin and a small amount of apigenin in its dark brown region, whereas M36001 accumulated only apigenin in its light brown region (**Figure 2**, A 290 nm). In summary, Nakei-MS3B accumulated 3-deoxyanthocyanidins and M36001 and #7 accumulated flavones, with different proportions of luteolin and apigenin.

**FIGURE 3:**
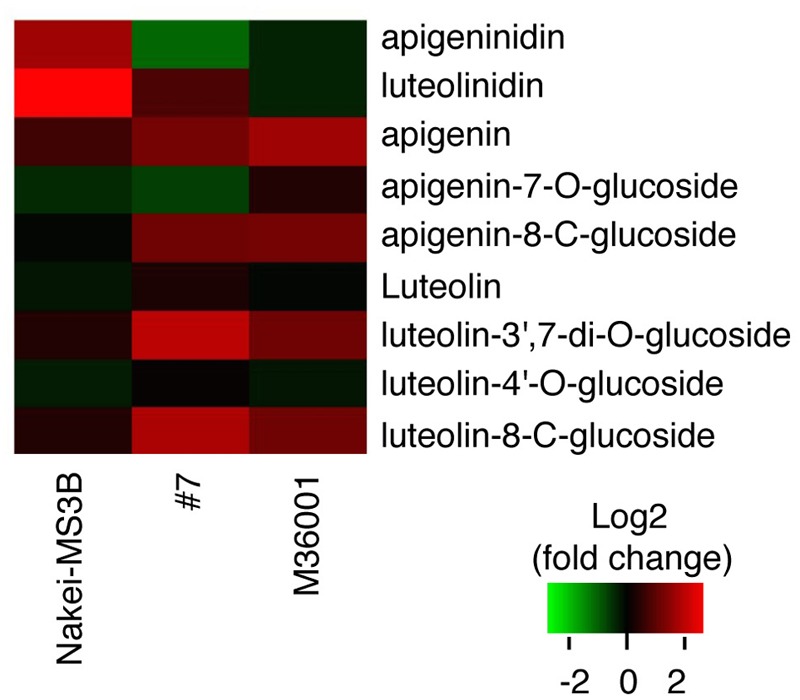
**Accumulation of glycosylated flavones.** Relative amount of glycosylated flavones is shown as a heatmap (red, upregulation; green, downregulation). Production of 3-deoxyanthocyanidins (luteolinidin and apigeninidin) was upregulated in Nakei-MS3B. Production of flavones and their derivatives was upregulated in M36001 and #7. Apigenin production was highly upregulated in M36001, and production of luteolin derivatives was highly upregulated in #7.

We then performed a metabolic analysis of the color pigments in each line by using liquid chromatography – mass spectrometry/mass spectrometry (LC-MS/MS) and standard authentic compounds (Sawada et al., 2009). The proportions of each compound are shown in the heatmap (**Figure 3**). Nakei-MS3B accumulated 3-deoxyanthocyanidins (luteolinidin and apigeninidin) after cutting stress. M36001 and #7 accumulated apigenin, apigenin-8-C-glucoside, luteolin-3′, 7-di-O-glucoside, and luteolin-8-C-glucoside. Apigenin was accumulated at high levels in M36001, whereas luteolin-3′, 7-di-O-glucoside, and luteolin-8-C-glucoside were accumulated at high levels in #7 (**Figure 3**).

**FIGURE 4:**
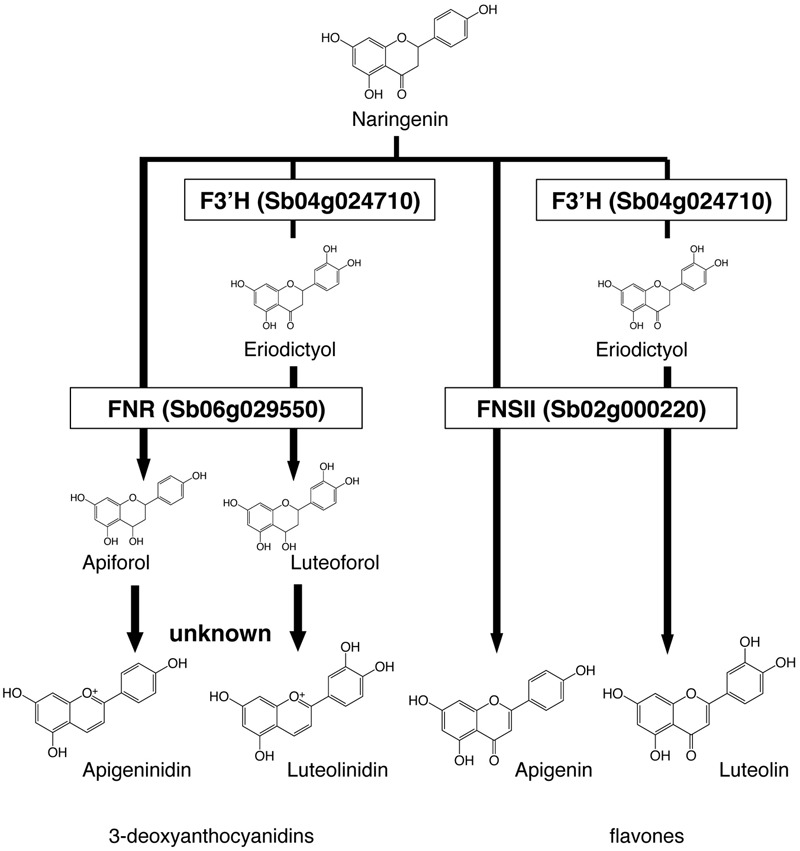
**Roles of *Flavanone 4-reductase (FNR), Flavone synthase II (FNSII)*, and *Flavonoid 3′-hydroxylase (F3′H)* in the metabolic pathways.** Naringenin is the common intermediate of 3-deoxyanthocyanidins and flavones. *FNR* converts flavanones (naringenin or eriodictyol) to flavan-4-ols (apiforol, luteoforol). *FNSII* converts naringenin to apigenin or eriodictyol to luteolin. *F3′H* introduces a hydroxyl group at the 3′ position of ring B of naringenin, adding the step that leads to the production of luteolinidin or luteolin.

We constructed a metabolic map based on our data and on the previous reports on enzyme activity (**Figure 4**). We considered F3′H, FNR, and FNSII play pivotal roles in the synthesis of these pigments. Apigeninidin, apigenin, luteolinidin, and luteolin are synthesized from a common intermediate, naringenin, through sequential reactions. F3′H hydroxylates the 3′ position of the B-ring of naringenin to produce eriodictyol; both are called flavanones (Boddu et al., 2004; Shih et al., 2006). FNR converts flavanones (naringenin or eriodictyol) to flavan-4-ols (apiforol or luteoforol, respectively), and this is followed by the synthesis of 3-deoxyanthocyanidins (apigeninidin or luteolinidin, respectively; Kawahigashi et al., 2016). FNSII converts flavanones (naringenin or eriodictyol) to flavones (apigenin or luteolin, respectively; Du et al., 2010).

### Structure and Expression of Genes Responsible for Color Variation

We assumed that the activation of each metabolic pathway (**Figure 4**) was controlled by the induction of gene expression. We performed RNA-seq to identify whole genes differentially expressed after cutting stress in each line (Supplementary Table S1) and made a schematic model of the relationship between the expressed genes and the pigments accumulated in each sorghum line. Here, we focused on *FNR* (Sb06g029550.1), *FNSII* (Sb02g000220.1), and *F3′Hs* (Sb04g024710.1, Sb04g024730.1, and Sb04g024750.1). The induction of each gene expression was confirmed by qRT-PCR.

(i) Nakei-MS3B (purple)

**FIGURE 5:**
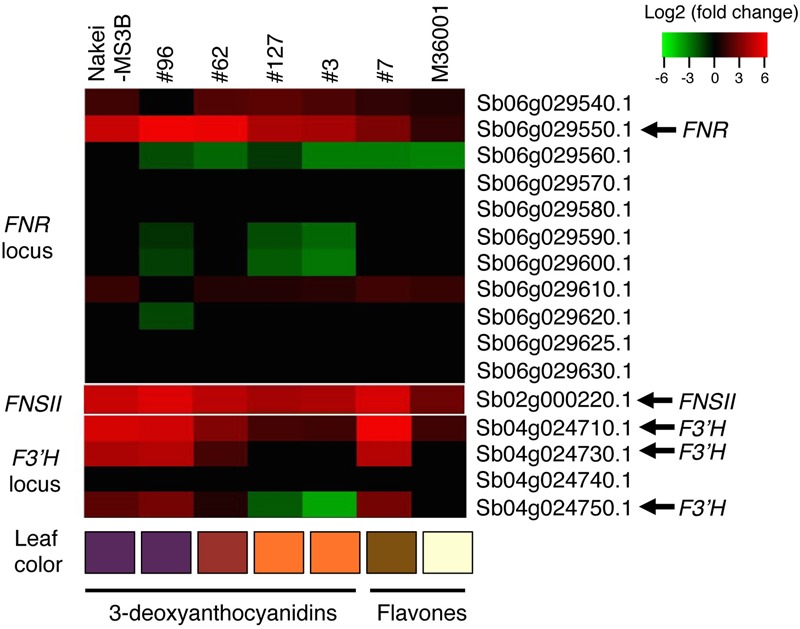
**Differential expression of *FNR, FNSII*, and *F3′H*.** Differentially expressed genes before and after cutting stress were identified by using RNA-seq. Log2 ratios of expression levels (4 days after cutting stress compared with immediately after cutting stress) of genes located at the *FNR, FNSII*, and *F3′H* loci are shown as a heatmap (red, upregulation; green, downregulation). Boxes at the bottom indicate colors of the cut edges of each sorghum leaf. *FNR (Sb06g029550.1)* expression suggests that this gene contributes to 3-deoxyanthocyanidin synthesis. Other tandemly arrayed homologous genes were similar to *FNR*, but their expression was not induced as strongly as that of *FNR*. Expression of *FNSII* was induced in all lines. The expression level of *F3′H* contributes to color variations determined by 3-deoxyanthocyanidins and flavones.

**FIGURE 6:**
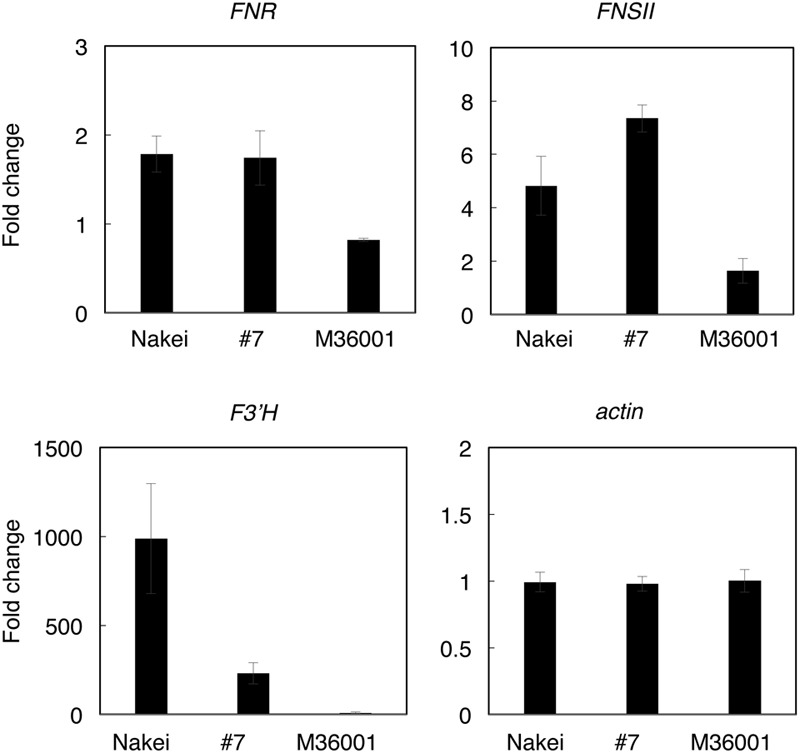
**Quantitative RT-PCR (qRT-PCR) analysis of genes involved in synthesis of apigenin, luteolin, apigeninidin, and/or luteolinidine.** Expression levels were quantified by qRT-PCR. Expression ratios (4 days after cutting stress compared with immediately after cutting stress) of genes for *FNR* (Sb06g029550), *FNSII* (Sb02g000220), *F3′H* (Sb04g024730), and *actin* (Sb03g040880) are shown. The expression level of each gene was normalized against that of the actin gene.

**FIGURE 7:**
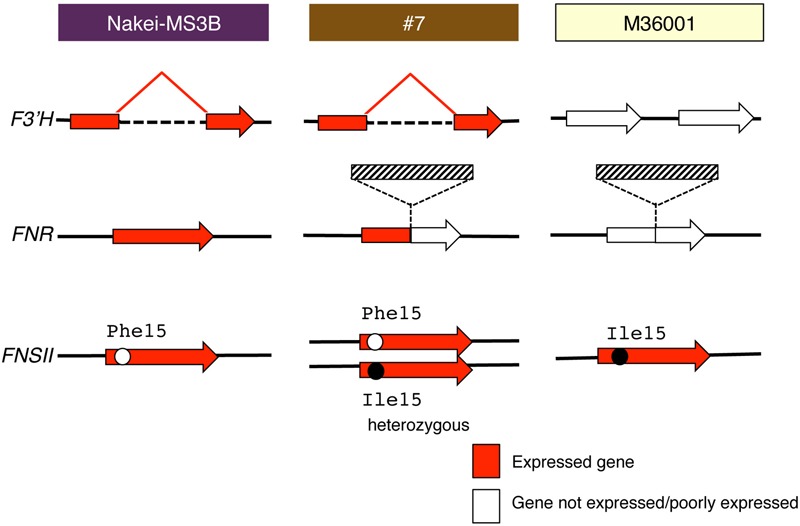
**Schematic structure and expression of *F3′H, FNR*, and *FNSII*.** Red arrows show gene expression; white arrows show that the gene is not expressed or is expressed very little. *F3′H* derived from Nakei-MS3B has a deletion and is highly expressed. *FNR* derived from M36001 has an insertion in the coding region; its expression is thus interrupted in #7. Hatched boxes show insertion in *FNR*. *FNSII* derived from Nakei-MS3B had Phe15, that from M36001 had Ile15, and that from #7 had heterozygous allele. The details of the gene structures and gene expression levels are shown in **Supplementary Figure S1**.

**FIGURE 8:**
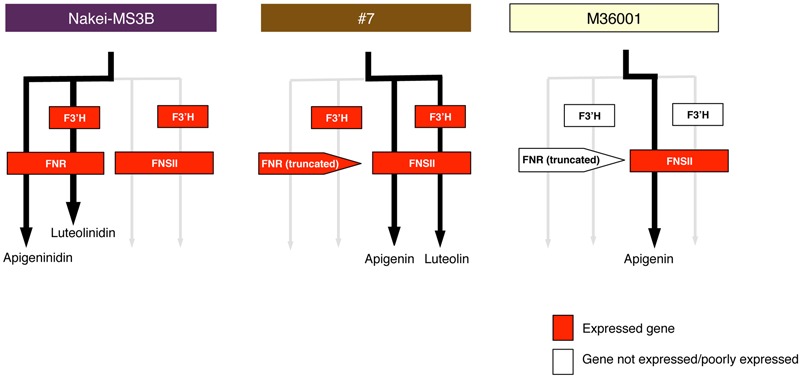
**Schematic models of accumulated pigments and expressed genes.** On the basis of the metabolome and transcriptome data, we show preferentially used metabolic pathways (black arrows) in Nakei-MS3B, M36001, and #7. Expressed genes are shown in red. Expression of *FNR* and *F3′H* led to synthesis of 3-deoxyanthocyanidins in Nakei-MS3B; expression of *FNSII* led to synthesis of apigenin (and its derivatives) in M36001; expression of *FNSII* and *F3′H* led to synthesis of apigenin and luteolin and their derivatives in #7.

Nakei-MS3B accumulated apigeninidin and luteolinidin in the purple region of its leaves (**Figures 1–3**). RNA-seq indicated that *FNR* (Sb06g029550.1) was upregulated among tandemly duplicated similar genes (Sb06g029540.1 to Sb06g029630.1; **Figure 5**, **Supplementary Figure S1**), suggesting that *FNR* production leads to the synthesis of 3-deoxyanthocyanidin (apigeninidin from naringenin). In addition, *F3′H* genes (Sb04g024710.1, Sb04g024730.1, and Sb04g024750.1) were upregulated (**Figures 5** and **6**, **Supplementary Figure S1C**), suggesting the occurrence of the additional step of hydroxylation of the 3′ position of the B-ring of naringenin, followed by the synthesis of luteolinidin. Thus, expression of *FNR* and *F3′H* led to the synthesis of 3-deoxyanthocyanidins (apigeninidin and luteolinidin) in Nakei-MS3B (**Figures 7** and **8**). This was consistent with a previous report that Nakei-MS3B and #96, #62, #127, #3 accumulated 3-deoxyanthocyanidins and showed injury-responsive color variations (purple, red, or orange) that depended on the expression level of *F3′H* genes (Mizuno et al., 2014).

(ii) M36001 (light brown)

M36001 accumulated apigenin and apigenin-8-C-glucoside in the light brown region of its leaves (**Figures 1–3**). *FNSII* was upregulated, but *FNR* and *F3′H* were not (**Figures 5** and **6**, **Supplementary Figure S1**), suggesting that expression of *FNSII* led to the synthesis of apigenin and its derivatives in M36001 (**Figure 8**).

(iii) #7 (dark brown, progeny of Nakei-MS3B and M36001)

#7 accumulated apigenin, luteolin, and their glucoside derivatives in the dark brown region of its leaves (**Figures 1–3**). Induction of the expression of *FNSII* and *F3′H* (**Figures 5** and **6**) thus likely led to the synthesis of these products in #7 (**Figure 8**). The *FNSII* allele was heterozygous: *FNSII* derived from Nakei-MS3B had Phe15, and that from M36001 had Ile15 (**Figure 7**). The *F3′H* gene was inherited from Nakei-MS3B, and highly expressed (**Figure 7**; **Supplementary Figure S1C**). The *FNR* gene in #7 inherited form M36001 had an insertion in the coding region at nucleotide 452 (**Figure 7**, **Supplementary Figure S1A**). Although the *FNR* gene was upregulated, this insertion divided the *FNR* gene and resulted in the production of truncated non-functional FNR protein (**Figures 7** and **8**).

### Searching for Putative Glucosyltransferase Genes in Sorghum

Our metabolic data suggested that glycosylated flavones were also accumulated in the leaves of #7 and M36001 (**Figure 3**). We used the RNA-seq data to search for a putative sorghum glucosyltransferase responsible for glycosylated flavone synthesis. Sb03g032050.1 was upregulated among the putative glucosyltransferase genes (**Supplementary Figure S2**); the protein it encoded has 73.9% amino acid identity with the functionally validated rice OsCGT (ABC94602/LOC_Os06g18010.1; Brazier-Hicks et al., 2009). We thus consider that the protein encoded by Sb03g032050.1. is a candidate glucosyltransferase involved in the synthesis of glycosylated flavones after injury.

## Discussion

### Expression of *FNSII* and *F3′H* is Associated with Colors in Tan-Colored Injured Leaves of sorghum

We aimed to elucidate the key genes for color variation in sorghum with leaves that turn tan upon injury. We identified accumulated pigments (**Figures 1–4**), determined the gene structure and expressed genes (**Figures 5–7**, Supplementary Table S1), and made a schematic model of a gene expression network for pigment synthesis (**Figure 8**). The expression of *FNSII, F3′H*, and/or *FNR* enables irreversible commitment to the metabolic pathway as they are located at the branch point from naringenin (**Figure 4**). We thus considered that the FNSII pathway was activated to synthesize flavones (apigenin and luteolin) and that the expression level of *F3′H* is associated with the balance of apigenin and luteolin, which in turn determined the dark or light brown variation in sorghums that respond to injury with tan pigmentation. Colors of extracted pigments from tan-colored sorghums were, however, different from those of the purified apigenin or luteolin monomers. One hypothesis is that dark brown or light brown pigments of tan-colored sorghum may be derived from polymers containing luteolin and/or apigenin, respectively. Moreover, glycosylation and other types of modifications of flavons (**Figure 3**) may change the colors of injured leaves of sorghum.

Here, among sorghum color variations (purple, red, orange, dark brown, and light brown), we focused on the color variation between dark brown (#7) and light brown (M36001). *F3′H* was highly expressed in #7; but not in M36001 and was consistent with luteolin accumulation (**Figures 2, 5, 6**, and **Supplementary Figure S1**). The high level of *F3′H* expression was likely due to the unique deletion in the *F3′H* locus inherited from Nakei-MS3B (**Figure 7**, **Supplementary Figure S1C**). We previously reported that other color variations (purple, red, and orange) are also determined by the level of *F3′H* expression, which results in differences in the proportions of 3-deoxyanthocyanidins (apigeninidin and luteolinidin; Mizuno et al., 2014). We therefore consider that expression of *F3′H* is associated with color variation in both the FNSII-dependent flavone pathway (light brown to dark brown) and the *FNR-*dependent 3-deoxyanthocyanidin pathway (purple to red to orange).

We speculate that at least two different transcription factors contributed to the expression of these color-related genes in sorghum: one for *FNSII* and the other for *F3′H* and *FNR*, because *FNSII* was upregulated in all lines used whereas *F3′H* and *FNR* was upregulated in Nakei-MS3B and #7, but not in M36001 (**Figure 7**; **Supplementary Figures S1A–C**). These results suggested M36001 lacks the functional transcription factor(s) that control injury-responsive expression of *F3′H* and *FNR*. As expression level of *F3′H* affected color variation in the *FNR* and *FNSII* pathways, the variation of these unknown transcription factors also have the potential to determine the injury-responsive color variation. As a candidate, *Yellow seed1* (*Y1*), which encodes an R2R3-MYB-type regulatory protein (Boddu et al., 2006), had 66.9% amino acid identity with maize *pericarp color1* (*p1*). P1 directly regulates the expression of *ZmF3′H*/*pr1* in the 3-deoxyflavonoid biosynthesis in maize (Grotewold et al., 1994; Sharma et al., 2011, 2012). However, *y1* (Sb01g037670) was not expressed with or without cutting stress in leaf (Supplementary Table S1). We therefore consider that regulation of color-related genes in sorghum differs between seed and leaf.

### Selection of Metabolic Pathways from Naringenin in Injury Response

Naringenin is the branching point of the FNR-dependent 3-deoxyanthocyanidin pathway and the FNSII-dependent flavone pathway (**Figure 4**). If both *FNR* and *FNSII* were substantially upregulated in Nakei-MS3B (**Figure 5**), why were only the downstream products of the FNR pathways detected (**Figure 2**)? One hypothesis is that, irrespective of *FNSII* expression, the FNR, rather than the FNSII, preferentially catalyzes the metabolism of the naringenin substrate. Another hypothesis is that amino acid substitution decreases the enzyme activity of FNSII, because FNSII in Nakei-MS3B had an Ile15Phe amino acid substitution (**Figure 7** and **Supplementary Figure S1B**). However, Ile15 was not conserved among rice, wheat, maize, and sorghum (Goodstein et al., 2012), suggesting that this substitution is not critical. In any case, we consider FNR-dependent pathway was dominant to FNSII-dependent pathway when both pathways were active. We thus consider that when the FNR-dependent pathway was not functional due to the insertion in *FNR* gene in #7 and M36001 (**Figure 7**), the FNSII-dependent pathway became dominant.

Naringenin is also the branching point of the anthocyanin pathway. The first product of the anthocyanin pathway from naringenin is synthesized by the action of flavanone 3-hydroxylase (F3H; Liu et al., 2010). However, in the tan-colored injury response we observed here, *F3H* (Sb06g031790.1) was not expressed (Supplementary Table S1), suggesting that the anthocyanin pathway was not activated; tan coloration was therefore independent of the production of anthocyanin metabolites. Our work expands scientific knowledge of the tan-colored injury response of sorghum cultivars—especially the gene expression behind this unique anthocyanin- and 3-deoxyanthocyanidin-independent coloration. Further comparative analysis will elucidate the diversity of the pigments accumulated and the gene expression network involved in the coloration of injured sorghums.

### Roles of Flavones in Sorghum

Sorghums accumulated flavones in their leaves (**Figure 2** in this study), grains (Dykes and Rooney, 2006), and glumes and sheaths (Siame et al., 1993). One of the roles of flavones is to act as phytoalexins. Several plant flavones have activity against a variety of organisms, including other plants, nematodes, mollusks, fungi, oomycetes, and bacteria (Martens and Mithofer, 2005). Although flavones are phytoalexins with broad specificity for organisms, their disease resistance activity depends on their molecular structures. For example, luteolin in sorghum is a stronger spore germination inhibitor than apigenin in *C. sublineolum* (Du et al., 2010). Flavone glycosides function as antimicrobial agents in *Thalassia testudinum* (Harborne and Williams, 2000). Thus, regulation of the genes involved in flavone synthesis might control disease resistance in sorghum: overexpression of *FNSII* might enhance the amount of flavones; overexpression of *F3′H* might shift accumulation from apigenin to luteolin; and overexpression of glucosyltransferase genes might promote the synthesis of glycosylated derivatives. Flavones also have antioxidant, anti-inflammatory, and anti-allergic properties (Seelinger et al., 2008) and inhibitory effects on human cancer cell proliferation (Zhang et al., 2005). Thus, flavones have potential for agrobiological, physiological, and therapeutic application.

## Conclusion

The dark or light brown color variation in injured leaves of sorghums is associated with the gene expression of *FNSII* and *F3′H.* We considered that FNSII pathway was activated to synthesize flavones (apigenin and luteolin) and that the expression level of *F3′H* changed the balance of apigenin and luteolin.

## Author Contributions

HKaw and SK prepared plant materials and performed cDNA synthesis; YS, MYH, and YO performed the metabolic analysis; HKan and TM performed the sequencing experiments; TY performed the data analysis; HM and HKaw designed the study; and HM wrote the manuscript. All authors read and approved the final manuscript.

## Conflict of Interest Statement

The authors declare that the research was conducted in the absence of any commercial or financial relationships that could be construed as a potential conflict of interest.

## Footnotes

1 https://cutadapt.readthedocs.org/en/stable/

2 http://www.R-project.org/

## Supplementary Material

FIGURE S1 **Expression of *Flavanone 4-reductase (FNR), Flavone synthase II (FNSII)*, and Flavonoid 3′-hydroxylase (*F3′H)*, and occurrence of single nucleotide polymorphisms (SNPs).** RNA-seq reads were mapped on the BTx623 reference genome and shown by using Integrative Genome Viewer. **(A)** *FNR*. *FNR* derived from M36001 has an insertion in the coding region; its expression is thus interrupted in #7. **(B)** *FNSII*. *FNSII* genes are expressed in all lines, but FNSII derived from Nakei-MS3B has two SNPs. One SNP results in amino I15F acid substitution (right arrowhead), but the other does not (left arrowhead). **(C)** *F3′H*. *F3′H* derived from Nakei-MS3B has a deletion and is highly expressed in Nakei-MS3B and #7.

FIGURE S2 **Differential expression of 10 putative C-glucosyl transferase genes.** Gene expression is shown as a heatmap (red, upregulation). Strong expression of Sb03g032050.1 was induced after cutting stress.
