# Supplementary figures and images for: Expression of Flavone Synthase II and Flavonoid 3′-Hydroxylase Is Associated with Color Variation in Tan-Colored Injured Leaves of Sorghum
Source: Front Plant Sci. 2016 Nov 21;7:1718. doi: 10.3389/fpls.2016.01718 (PMC5116553; doi:10.3389/fpls.2016.01718)

*FNR*

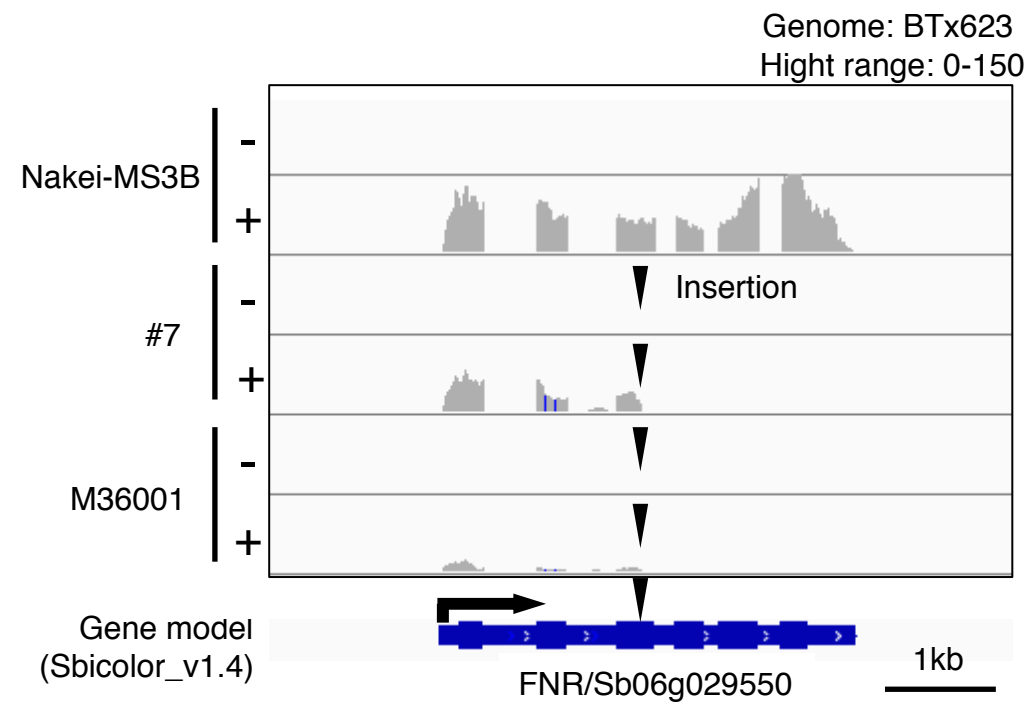

Fig. S1A

*FNSII*

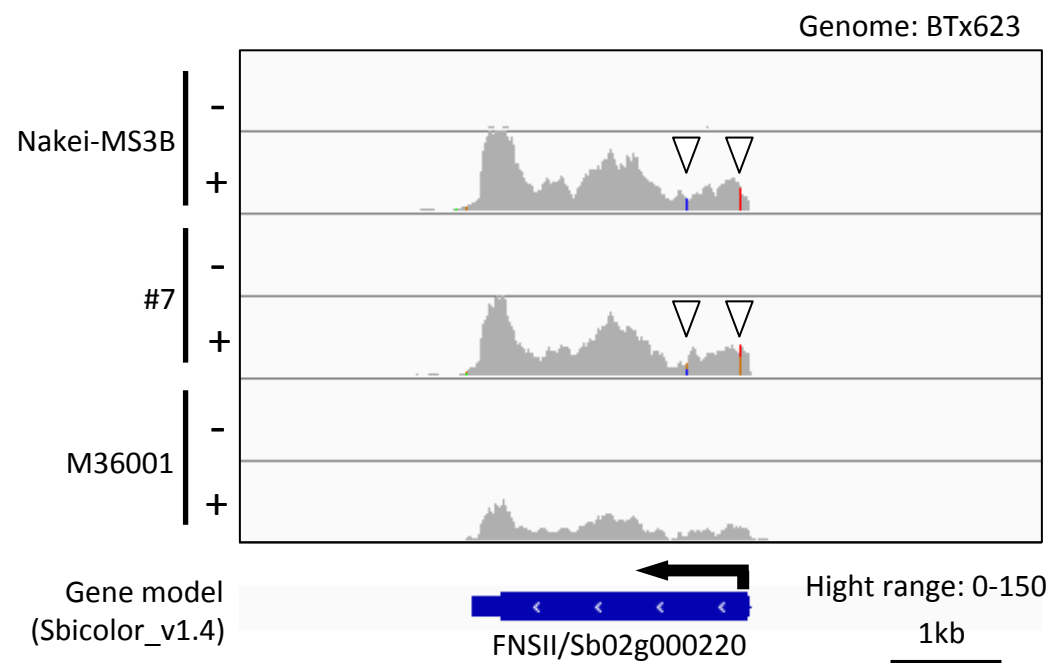

Fig. S1B

*F3'H*

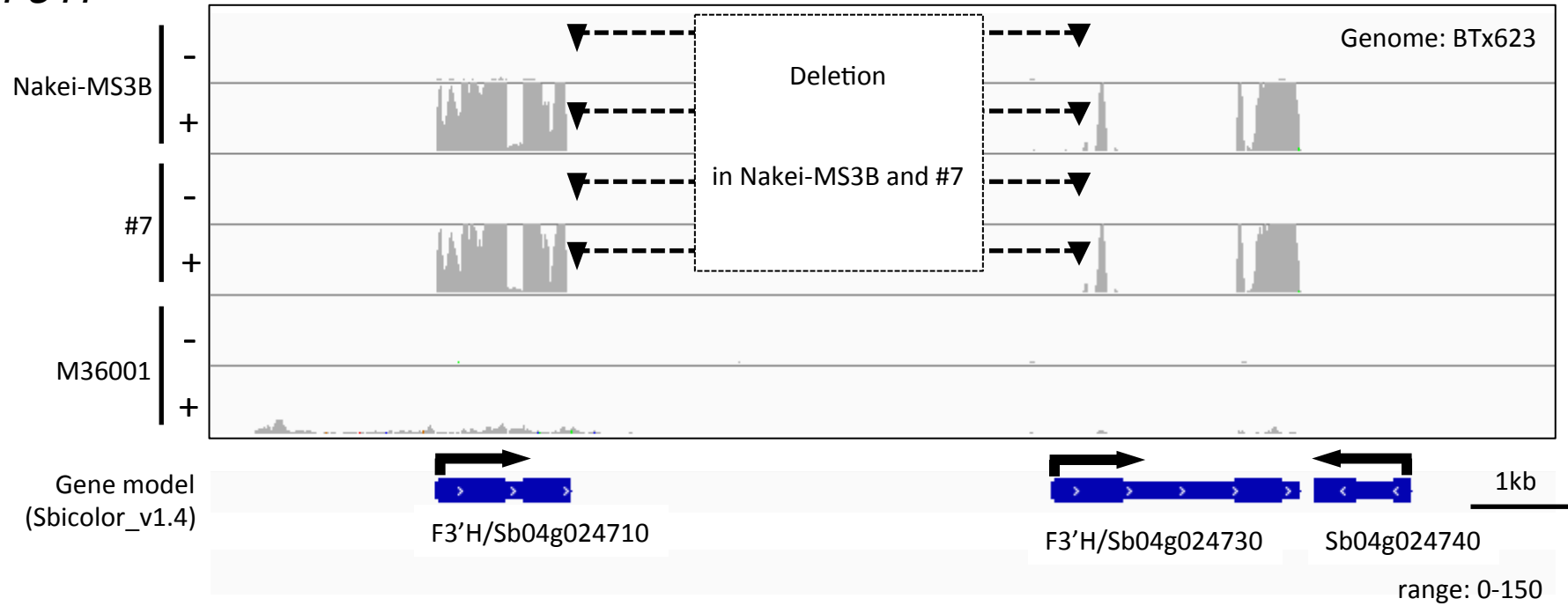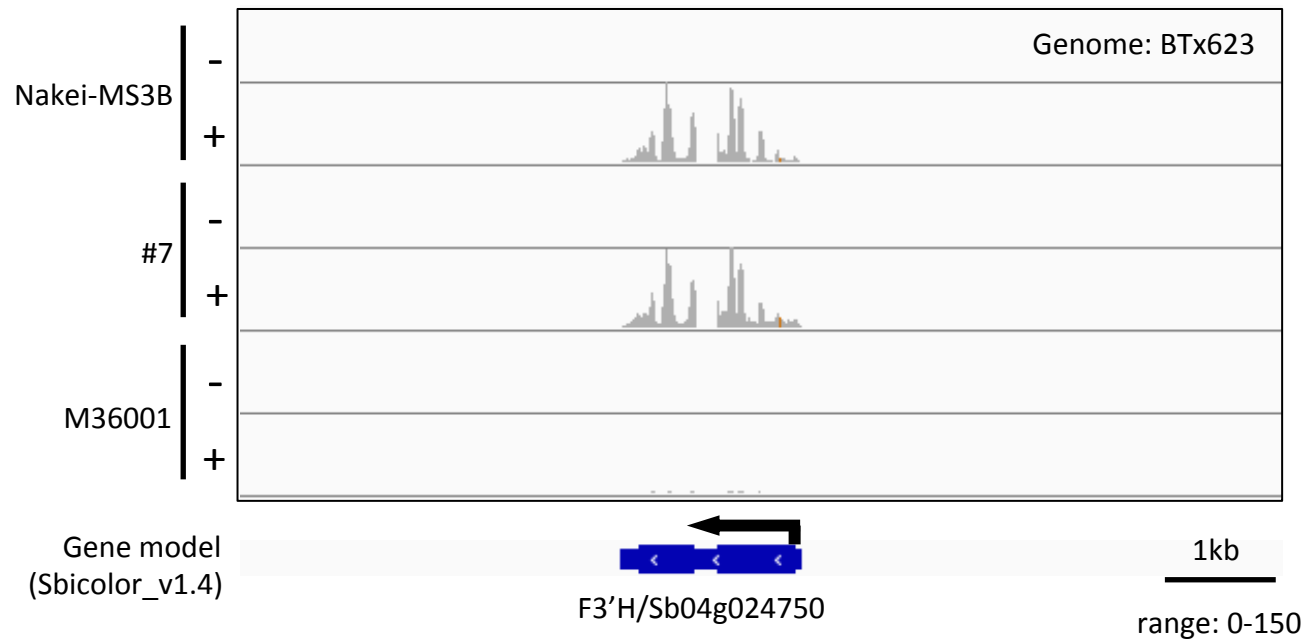

Fig. S1C

Supplement: FIGURE S1 — Expression of Flavanone 4-reductase (FNR), Flavone synthase II (FNSII), and Flavonoid 3′-hydroxylase (F3′H), and occurrence of single nucleotide polymorphisms (SNPs). RNA-seq reads were mapped on the BTx623 reference genome and shown by using Integrative Genome Viewer. (A) FNR. FNR derived from M36001 has an insertion in the coding region; its expression is thus interrupted in #7. (B) FNSII. FNSII genes are expressed in all lines, but FNSII derived from Nakei-MS3B has two SNPs. One SNP results in amino I15F acid substitution (right arrowhead), but the other does not (left arrowhead). (C) F3′H. F3′H derived from Nakei-MS3B has a deletion and is highly expressed in Nakei-MS3B and #7. [file Image_1.PDF]

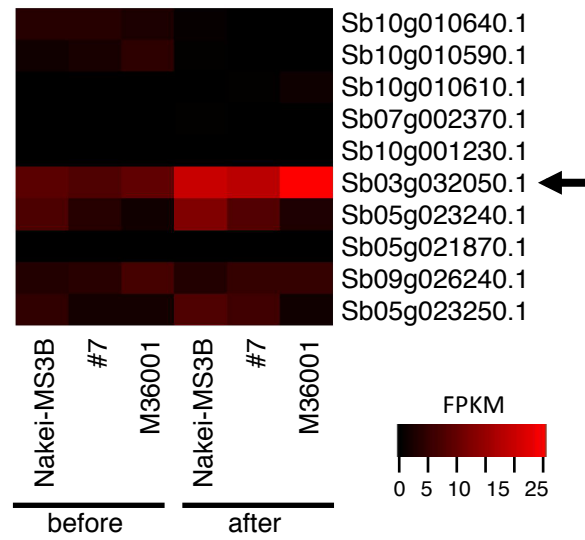

Supplement: FIGURE S2 — Differential expression of 10 putative C-glucosyl transferase genes. Gene expression is shown as a heatmap (red, upregulation). Strong expression of Sb03g032050.1 was induced after cutting stress. [file Image_2.PDF]
